# Differences in the Signaling Pathways of α_1A_- and α_1B_-Adrenoceptors Are Related to Different Endosomal Targeting

**DOI:** 10.1371/journal.pone.0064996

**Published:** 2013-05-24

**Authors:** Vanessa Segura, Miguel Pérez-Aso, Fermí Montó, Elena Carceller, María Antonia Noguera, John Pediani, Graeme Milligan, Ian Christie McGrath, Pilar D’Ocon

**Affiliations:** 1 Departamento de Farmacología, Facultad de Farmacia, Universitat de València, Valencia, Spain; 2 Molecular Pharmacology Group, Institute of Molecular, Cell and Systems Biology, College of Medical, Veterinary and Life Sciences. University of Glasgow, Glasgow, United Kingdom; 3 Autonomic Physiology Unit, School of Life Sciences, College of Medical, Veterinary and Life Sciences, University of Glasgow, Glasgow, United Kingdom; University of North Dakota, United States of America

## Abstract

**Aims:**

To compare the constitutive and agonist-dependent endosomal trafficking of α_1A_- and α_1B-_adrenoceptors (ARs) and to establish if the internalization pattern determines the signaling pathways of each subtype.

**Methods:**

Using CypHer5 technology and VSV-G epitope tagged α_1A_- and α_1B_-ARs stably and transiently expressed in HEK 293 cells, we analyzed by confocal microscopy the constitutive and agonist-induced internalization of each subtype, and the temporal relationship between agonist induced internalization and the increase in intracellular calcium (determined by FLUO-3 flouorescence), or the phosphorylation of ERK1/2 and p38 MAP kinases (determined by Western blot).

**Results and Conclusions:**

Constitutive as well as agonist-induced trafficking of α_1A_ and α_1B_ ARs maintain two different endosomal pools of receptors: one located close to the plasma membrane and the other deeper into the cytosol. Each subtype exhibited specific characteristics of internalization and distribution between these pools that determines their signaling pathways: α_1A_-ARs, when located in the plasma membrane, signal through calcium and ERK1/2 pathways but, when translocated to deeper endosomes, through a mechanism sensitive to β-arrestin and concanavalin A, continue signaling through ERK1/2 and also activate the p38 pathway. α_1B_-ARs signal through calcium and ERK1/2 only when located in the membrane and the signals disappear after endocytosis and by disruption of the membrane lipid rafts by methyl-β-cyclodextrin

## Introduction

There is evidence that for the α_1_-ARs the constitutive and agonist-driven cellular internalization differs between α_1A-_ and α_1B-_ subtypes. This could have important implications for the different physiological roles of these subtypes and for the therapeutic use of selective antagonists of this receptor sub-family. However, there are contradictions in what these differences are between studies conducted using different techniques. Since this could be important for the physiology of the receptors and for the pharmacology and therapeutic use of subtype-selective antagonist ligand drugs (α_1-_ blockers) in the cardiovascular and urogenital systems, we have taken a new technical approach to the problem in an attempt to resolve this controversy.

α_1_-Adrenoceptors (α_1_-ARs) are heptahelical transmembrane proteins that belong to the G protein-coupled receptor (GPCR) superfamily [1]. Upon agonist binding, all three α_1_-AR couple to the Gq/11 protein [2], resulting in phosphatidylinositol 4,5-biphosphate hydrolysis via phospholipase Cβ activation; resultant second messengers include inositol 1,4,5-triphosphate and diacylglycerol, which mobilize intracellular calcium and activate protein kinase C, respectively [3], [4], [5]. Many lines of evidence suggest that activation of α_1_-ARs also induce phosphorylation of mitogen-activated protein kinases (MAPKs) as p38 and extracellular signal-regulated kinase (ERK) [6], [7], [8]. The G-protein mediated signaling of α_1_-ARs requires that receptors, G_q/11_ proteins and phospholipase C are located in the plasma membrane. Termination of this pathway occurs by desensitization and endocytosis of the GPCRs, which involves phosphorylation of the activated receptor by G-protein-coupled Receptor Kinases (GRKs) and second-messenger-dependent protein kinases PKA and PKC [9], [10], and subsequent recruitment of the multifunctional adapter protein β-arrestin.

It has long been known that β-arrestin prevents G protein signaling by physically uncoupling the interaction between a GPCR and its associated G protein, and initiates clathrin-mediated internalization, facilitating the formation of the clathrin coated pits required for receptor endocytosis. Following endocytosis, the receptors traffic through divergent endosomal pathways lead to different fates. Some are resensitized by dephosphorylation, and recycled back to the cell surface. Others undergo down-regulation by being targeted to lysosomes where they are degraded. Some GPCRs remain intact and continue to signal, or initiate new signaling pathways independent of arrestins [11].

The best studied example of an arrestin-activated signaling pathway is the ERK cascade. Recent studies have shown that adrenoceptors can initiate ERK signaling by both G-protein- and β-arrestin-dependent processes [12], [13]. β-arrestins function as membrane-tethered scaffolds capable of recruiting elements of the MAPK pathways to membranes of endosomes, thus facilitating ERK activation. Furthermore, by anchoring activated ERK to endosomes, β-arrestins might prevent ERK translocation to the nucleus, thus favoring cytoplasmic ERK signaling [14], [15], [16], [17], [18]. Interestingly, the time course and molecular consequences of activating ERK signaling through G-protein mediated pathways versus β-arrestin mediated pathways are considerably different [13].

A few studies have investigated the internalization properties of the α_1_-AR subtypes with divergent conclusions. Following agonist stimulation, α_1B_-AR is rapidly desensitized by GRKs and its clathrin-mediated internalization involves β-arrestin interaction [19], [20], [21]. This subtype undergoes constitutive internalization according to Stanasila et al. [22] but not according to Morris et al. [23]. The most intriguing results have been observed with the α_1A_-AR. This subtype has been observed in lipid rafts under basal conditions [24], [25] and, according to some authors, undergoes constitutive and phenylephrine (PHE)-mediated internalization via clathrin-coated vesicles [23], [26], [27]. In contrast, other authors [22] did not find constitutive internalization for this subtype possibly due to differences in the expression system (stable *vs* transient), the receptor constructs (GFP-tagged *vs* HA-tagged) or the methods used to measure endocytosis (fluorescent images techniques *vs* biotinylation experiments).

The aim of the present work was to explore the constitutive and agonist-dependent endosomal trafficking of α_1A_- and α_1B-_ARs, using CypHer5 technology [28], [29] and VSV-G epitope tagged receptors stably and transiently expressed in HEK 293 cells. In order to establish if the internalization pattern determines the signaling pathways and explains differences in the functional role of each subtype, we also analyzed the temporal relationship between internalization and the increase in intracellular calcium, a signal directly related to the interaction of ARs with the G-protein in the membrane, as well as intracellular signals not necessarily dependent on G proteins, such as activation of MAPKs (ERK1/2 and p38).

## Experimental Procedures

### 1. Multiple Fluorescent Labeling of Recycling and Late Endocytic Vesicles in unstimulated Rat-1 fibroblasts

Rat-1 fibroblasts, (R-1Fs), stably expressing the human α_1A_ or α_1B_-AR, grown on sterile coverglasses were rinsed with PBS and then exposed at room temperature to PBS containing 10 nM QAPB and either the recycling endosomal marker transferrin, (Tfn-)-Alexa Fluor^ 546^ (20 µg/ml) or the late endosomal/lysosomal marker Lysotracker Red DND-99 (150 nM) for 15 and 70 min, respectively. At the end of each labeling period, cells were rinsed with PBS supplemented only with QAPB (10 nM). Approximately 10 min before image acquisition, cells were exposed to PBS containing 10 nM QAPB plus the nuclear DNA-binding dye Hoechst 33342 (10 µg/ml; 10-min incubation at room temperature) to stain cell nuclei. Each coverglass was then washed and bathed in QAPB containing PBS before being imaged. The dyes Hoechst 33342, QAPB, transferrin, (Tfn), Tfn-Alexa Fluor^546^, and Lysotracker Red were sequentially excited using the appropriate fluorescence filter sets to prevent bleed through, and the resultant images were overlaid using MetaMorph software (version 7.7.7, Molecular Devices, Sunnydale, CA). 3D visualisation of the colocalisation of QAPB ligand-human α_1a_ or α_1b_-AR complexes with Lysotracker Red was achieved by using a Nikon TE2000-E inverted microscope equipped with a *z*-axis linear encoded stepper motor. Each dye was sequentially excited, and a z-series of images was acquired at 0.22-µm steps to produce individual z-stacks. The z-stack images were then merged and deconvoluted using an iterative and constrained algorithm (Autodeblur software, version 9.3.6; Autoquant Imaging, Media Cybernetics, Inc., Silver Spring,). A 3D x-z maximum projection image was constructed using Autovisualise software (Autoquant Imaging, Media Cybernetics, Inc., Silver Spring, MD).

For the analysis of QAPB-labeled human α_1A_ or α_1B_ -AR, with Tfn-Alexa Fluor546 or Lysotracker Red, a region of no fluorescence, (black area), adjacent to the cell was used to determine the average background level of autofluorescence average in each channel image acquired. This background autofluorescence amount was then subtracted from each pixel in each channel image collected. Pearson correlation coefficients that described the degree by which QAPB and Lysotracker Red fluorescence varied from a perfect correlation overlap value of 1.00 were measured by comparing the amounts of fluorescence measured in each matched pixel of the two different channel images acquired.

The degree of partial colocalisation detected when QAPB-labeled human α_1A_ or α_1B_-AR fused with Tfn-Alexa Fluor546 labeled recycling endosomes was quantified from a rectangular region of interest (ROI) drawn and superimposed on each channel image in exactly the same x-y position. Using the Metamorph “correlation plot” module, correlation coefficients were quantified that described the degree by which QAPB and Tfn-Alexa Fluor546 fluorescence at each pixel within the rectangular region varied from a perfect correlation of 1.00.

### 2. Construction of the VSV-G-Tagged α_1_-AR subtypes

VSV-G (YTDIEMNRLGK)epitope tags were introduced immediately upstream of each human α_1A_-, and α_1B_-AR. The amino-terminal primer (5′- AAAAAAAGGATCCGCCACCATGTACACCGATATAGAGATGAACAGGCTGGGAAAGGTGTTTCTCTCGGGAAATGC-3′) or (5′-AAAAAAAGCTTCCACCA TGTACACTGATATCGAAATGAACCGCCTGGGTAAGAATCCCGACCTGGACACCG-3′) was used to incorporate a VSV-G tag, a kozak sequence and a BamHI, HindIII and HindIII site for α_1A_-, and α_1B_-AR, respectively. Depending on the subtype used as a template, the following carboxyl-terminal reverse primer was hybridized: α_1A_-AR, 5′-AAAAAAAACTCGAGCTAGACTTCCTCCCCGTTC-3′ and α_1B_-AR, 5′-AAAAAGAATTCCTAAAACTGCCCGGGCGC-3′, incorporating a XhoI, EcoRI and EcoRI site downstream of the coding sequence. Oligonucleotides were purchased from Thermo Electron corporation (Glasgow, United Kingdom). All the PCR fragments were subsequently cloned into the plasmid pcDNA3 (Invitrogen, Paisley, Renfrewshire, Scotland, UK) in-frame ligation. Construct sequences were confirmed by nucleotide sequencing.

### 3. Cell Culture and Transfection

HEK293 cells were grown in Dulbecco’s modified Eagle’s minimum essential medium (Sigma St Louis MO, USA) supplemented with 10% newborn calf serum (Gibco BRL, Gaithersburg, MD, USA), L-glutamine 2 mM (Gibco BRL, Gaithersburg, MD, USA), 100 µg/ml streptomycin and 100 units/ml penicillin at 37°C in a humidified atmosphere of 5% CO_2_. Cells were stably transfected by electroporation (300 V, 50 µs, 2 mm gap) using a Multiporator (Eppendorf AG, Hamburg, Germany) After 48h, cells were selected with G418 400 µg/ml (Sigma, St Louis MO, USA). All the experiments were performed with around 75% of confluence. For transient transfection, HEK293 cells were grown to 50% confluence prior to transfection on poly (L-lysine)-coated coverslips without using antibiotics. Transient transfection was performed using Lipofectamine reagent (Invitrogen) according to the manufacturer’s instructions. A total of 1 µg of pcDNA3 containing the appropriate VSV-G tagged human α_1_-AR subtype was used to transfect each coverslip.

Quantitative reverse-transcription polymerase chain reaction and saturation binding experiments performed as previously described [30] confirmed the stable or transient expression of each subtype of VSV-G-tagged or untagged α_1_-ARs in the HEK293 cell lines.

### 4. Real-time imaging of the internalization of the α_1_-AR subtypes

HEK293 cells transiently or stably expressing the N-terminal VSV-G tagged human α_1A_- and α_1B_-AR subtypes were plated onto poly-L-Lysine (Sigma, St Louis MO) coated sterile coverslips 48h before experimentation. Live cells were washed three times with cold Krebs-Ringer-Hepes buffer (KRH, 120 mM NaCl, 25 mM HEPES, 4.8 mM KCl, 1.2 mM MgSO_4_ and 1.3 mM CaCl_2_ at pH 7.4) at 4°C and were then incubated with CypHer5E Linked Anti-VSV Antibody (PA45407, GE Healthcare, Amersham International, Buckinghamshire, UK) at 5 µg/ml in KRH buffer at 4°C for 1 h. After washing with KRH buffer at 4°C, coverslips were rapidly mounted into a flow chamber bath (Attofluor, Molecular Probes; Eugene, OR, USA), placed on the microscope stage in a 95% air and 5% CO_2_ atmosphere at 37°C. At this time, cells were exposed to prewarmed KRH buffer or buffer supplemented either with concanavalin A (ConA, 250 µg/ml, Sigma St Louis MO, USA) or prazosin (10 µM, Sigma St Louis MO, USA) for 30 min. After incubation with the aforementioned solutions, the zero time was set up immediately before adding phenylephrine (PHE, 100 µM, Sigma St Louis MO, USA) and the system was set to acquire images at 1 minute and then at 5 min intervals for 15 min. A set of control experiments without agonist was performed in the same conditions to analyze constitutive internalization. A laser-scanning confocal inverted microscope (LEICA TCS SP2 (DM-IRBE), equipped with a 60x oil HCX PL APO (1.32 numerical aperture) objective, was used to acquire images. The excitation wavelength was 633 nm using a Helium/Neon laser and the emitted fluorescence was detected with a 650 nm long pass filter. Image processing and analysis were carried out using MetaMorph, version 6.1r3 (Universal Imaging Corporation) and the Leica software, v. 2.61. By considering that all the fluorescence obtained was due to endosomal localization of receptors, two distinct fluorescence regions were defined in the same cell: a fraction close to the cytoplasmic side of the plasma membrane and another fraction achieving the profound cytosolic regions close to the nuclei. To identify membrane and cytosolic regions more clearly, light microscopic images were overlapped with fluorescence images. Internalization kinetic was quantified for each cell at these two different cellular regions in 8-10 different cells for each experiment, by measuring the mean intensity of the fluorescence of two linear segments of 5 µm of length located in the cytosol, close to the nucleus, and two linear segments of 5 µm of length located in regions close to the plasma membrane (Figure S1). To better identify membrane and cytosolic regions, light microscopic images were overlapped with fluorescence images.The mean of these determinations was normalized as I_i_/I_0_, where I_0_ is the mean intensity taken at zero time for each experiment. The data represent more than four independent experiments.

### 5. Real-time imaging of calcium signal

To monitor intracellular calcium concentration, HEK293 cells stably expressing the N-terminal VSV-G tagged or untagged human α_1A_- and α_1B_-AR subtypes were plated onto poly-L-Lysine coated sterile coverslips 48h before experimentation, washed three times with cold Krebs-Ringer-Hepes buffer (KRH, 120 mM NaCl, 25 mM HEPES, 4.8 mM KCl, 1.2 mM MgSO_4_ and 1.3 mM CaCl_2_ at pH 7.4) at 4°C and incubated for 2 hours with the fluorescent calcium chelator FLUO-3-AM (5 µM) (Invitrogen, Carlsbad CA, USA) in KRH at 5% CO_2_ and 37°C. Then, cells were washed once with KRH and mounted into a flow chamber bath placed on the microscope stage in a 95% air and 5% CO_2_ atmosphere at 37°C as has been described above. At this time, cells were exposed to prewarmed KRH buffer or buffer supplemented with prazosin (10 µM). Following 30 minute incubation with the aforementioned solutions, the zero time was set up immediately before adding PHE 100 µM and the system was set to acquire images at 1minute and then at 5 minute intervals for 15 min. A 63x oil HCX PL APO (1.32 numerical aperture) objective, was used to acquire images. The excitation wavelength was 488 nm using an Argon laser and the emitted fluorescence was detected with a 500–550 nm band-pass filter. We performed experiments in parallel with and without PHE in presence or absence of 10 µM prazosin to determine the spontaneous changes in the fluorescence due to the experimental procedure. As a slight but continuous increase in the fluorescence intensity was observed in the absence of PHE, in order to avoid non specific fluorescence, the increased calcium signal elicited in absence of PHE or in the presence of prazosin was subtracted from the PHE calcium signal.

### 6. Preparation of Cellular Extracts and Immunoblotting

HEK293 cells were starved for 4h in serum-free medium in presence or absence of methyl-β-cyclodextrin (mβCD, 10 mM, Sigma, St Louis MO, USA) for 30 or 60 min and filipin 1 µg/ml, prazosin 10 µM or 5-methylurapidil 10 µM for 30 min, followed by stimulation with PHE 100 µM for a 15 min time course at 37°C. After stimulation, cells were washed once with cold PBS and lysed by rotating 30 min at 4°C with 500 µl of RIPA buffer (50 mM HEPES, 150 mM NaCl, 10% glycerol, 1.5 mM MgCl_2_, 0.1% SDS, 1 mM EGTA, 1% triton and 1% sodium deoxycholate) containing protease inhibitor cocktail (Complete®, Roche Applied Science, Germany) and phosphatase inhibitor cocktail (PhosSTOP®, Roche Applied Science, Germany) followed by sonication with a Microson™ XL 2000 Ultrasonic Liquid Processor and storage at -80°C. The protein content was measured by the Bradford (1976) method (Bio-Rad Hercules CA, USA). 15 µg of cellular extracts were incubated with SDS-sample buffer (2% SDS, 60 mM Tris buffer, 5% β-mercaptoethanol, 0.01% bromophenol blue and 10% glycerol) at 40°C during 30 min, separated on 10% SDS-polyacrylamide gels and transferred to PVDF membranes for immunoblotting. Prior to antibody incubation, membranes were blocked in phosphate-buffered saline with 0.1% Tween 20 (PBST) plus 3% BSA (Albumin from bovine serum, Sigma St Louis MO, USA). Anti phospho-p42/44 ERK MAPK (Thr202/Thr204), p42/44 ERK MAPK (Cell Signaling Technology, Beverly, MA), p-p38 Anti-ACTIVE® p38 pAb, Rabbit, (pTGpY) (Promega Corp., Madison USA) and p38 (Cell Signaling Technology, Beverly, MA) antibodies were incubated overnight at 4°C, 1/500 diluted, while anti actin (Sigma St Louis MO, USA) antibody was used at a 1/2500 dilution. Membranes were then washed three times with PBST, incubated with antirabbit IgG horseradish peroxidase-conjugated secondary antibody (Amersham International, Buckinghamshire, UK) at 1∶2500 (Amersham Biosciences, UK) for 45 min at room temperature and washed extensively with PBST before chemiluminescent detection was performed using the ECL Western Blotting Detection Reagents (Amersham International, Buckinghamshire, UK). The image was captured with the AutoChemi System (uvp Bioimaging Systems. Cambridge, UK) and band intensity was measured using LabWorks Image acquisition and Analysis (uvp Bioimaging Systems. Cambridge, UK).

### 7. Statistical Analysis

The results are presented as the mean ± S.E.M. for n independent experiments performed in different days. A statistical analysis was performed by two-way ANOVA or by the Student’s *t* test for unpaired samples (GraphPad Software, Inc. San Diego CA, USA). Significance was defined as p<0.05.

## Results

### α_1A_- and α_1B_-ARs both locate with early and late endosomes

**Figure 1:**
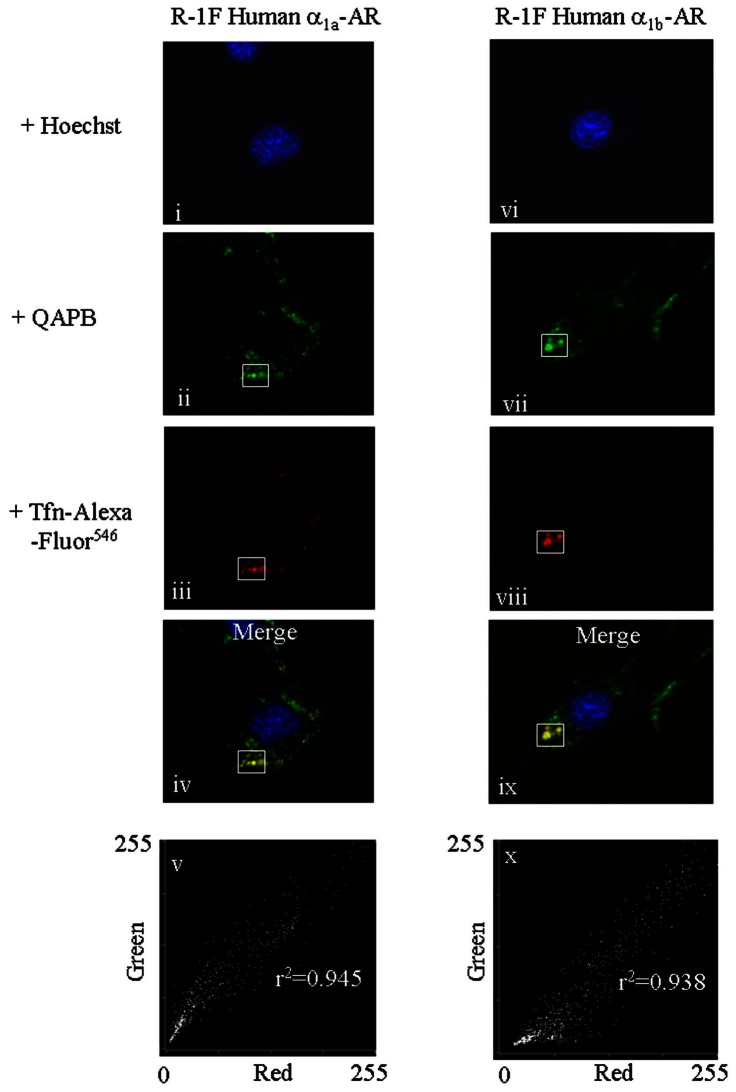
Internalised QAPB ligand-α_1A_-AR and α_1B_-AR complexes colocalize with the recycling endosomal fluorescent ligand marker Transferrin, (Tfn), -Alexa Fluor546 in unstimulated living R-1Fs stably expressing the human α_1A_ -AR or α_1B_-AR. In image i, nuclei stained with Hoechst are shown in blue. ii, green punctates represent QAPB (10 nM)-labeled human α_1A_ -ARs, and iii, recycling vesicles labeled with Tfn-Alexa Fluor546 are represented by the red punctate spots. Overlay image iv shows the partial colocalization (yellow vesicles) detected when unstimulated R-1Fs stably expressing α_1A_ -AR-QAPB ligand complexes fuse with the recycling red fluorescent endosomal marker. Red-Green pixel scatter intensity plots constructed from the intensity values located within the rectangular region superimposed on each channel image ii–iii in exactly the same x–y position is illustrated in v and r2 represents the Pearson overlap correlation coefficient value quantified from the matched regions defined in image ii and iii. Blue color in image vi represents Hoechst stained nuclei. vii, green punctates represent QAPB (10 nM)-labeled human α_1B_-ARs, and viii, recycling vesicles labeled with fluorescent Tfn are represented by the red punctate spots. Merge image ix shows the partial colocalization (yellow vesicles) observed when α_1B_-AR-QAPB ligand complexes fuse with the recycling red fluorescent endosomal marker. Pearson correlation coefficient value, (r2), measured from the intensity values located within the rectangular region superimposed on images vii–viii is illustrated in image ix.

**Figure 2:**
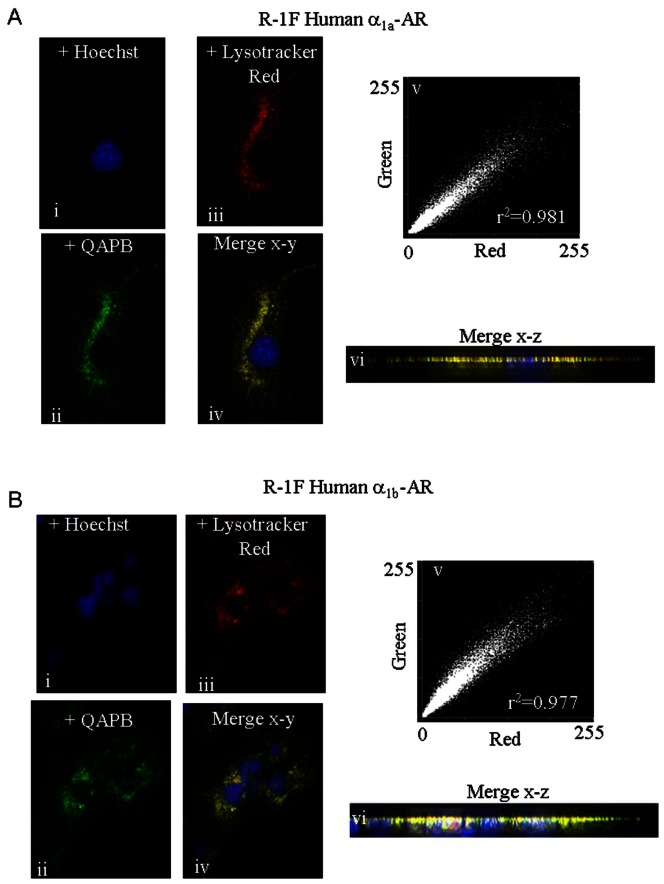
Internalised QAPB ligand- α_1A_-AR and α_1B_-AR complexes colocalize with the late endosomal fluorescent marker Lysotracker Red DND-99 in living R-1Fs stably expressing the human α_1A_-AR or α_1B_-AR. (A). In image i, nuclei stained with Hoechst are shown in blue. ii, green punctates represent QAPB (10 nM)-labeled human α_1A_ -ARs, and iii, acidic late endosomes labeled with Lysotracker Red DND-99 for 80 minutes are represented by the red punctate spots. Overlay image iv shows the extensive colocalization (yellow vesicles) detected when unstimulated R-1Fs stably expressing α_1A_ -AR-QAPB ligand complexes fuse with the late endosomes fluorescently labeled with Lysotracker Red. Linear red-green pixel scatter intensity plots constructed from the intensity values located within images ii–iii is illustrated in v and r2 represents the Pearson overlap correlation coefficient value quantified from each channel image ii and iii. 3D x-z colocalization maximum projection view of human α_1A_ -AR-QAPB ligand complexes fusing with late/lytic endosomes is shown in iv (yellow punctates).(B). In image i, nuclei stained with Hoechst are shown in blue. ii, green punctates represent QAPB (10 nM)-labeled human α_1B_-ARs, and iii, late endosomes labeled with Lysotracker Red for 80 minutes are illustrated by the red punctate spots. Merge image iv shows the extensive colocalization (yellow vesicles) detected when unstimulated R-1Fs stably expressing α_1B_-AR-QAPB ligand complexes fuse with the late endosomes fluorescently labeled with Lysotracker Red. Linear green-red pixel scatter intensity plots constructed from the intensity values located within images ii–iii is illustrated in v and r2 represents the Pearson overlap correlation coefficient value quantified from each channel image ii and iii. A 3D x-z colocalization maximum projection view of human α_1B_-AR-QAPB ligand complexes interacting with late/lytic endosomes is shown in iv (yellow punctates).

A short 15 min exposure to the recycling endosomal marker transferrin, (Tfn-)-Alexa Fluor^ 546^ produced its colocalization with the fluorescent ligand QAPB in compartments near to the cell membrane for both α_1A_- and α_1B_-ARs indicating transition of the receptors through this compartment (Figure 1). Similarly, for both α_1A_- and α_1B_-ARs a more prolonged exposure of 70 min to the late endosomal/lysosomal marker Lysotracker Red DND-99 indicated its colocalization with QAPB in a much greater number of endosomes located throughout the cytoplasm and as deep as the nuclear membrane (Figure 2). Together this indicates that the recycling receptors enter the near-membrane endosomal compartment and subsequently populate the deeper endosomal compartments.

### Constitutive internalization and intracellular distribution patterns of α_1_-ARs are subtype-specific

We analyzed the internalization kinetics of the α_1_-ARs, as well as the specific subcellular distribution of each subtype related to its internalization kinetics. For this purpose, the VSV-G tag was inserted into the amino terminal sequence of the α_1A_- and α_1B_-ARs that had been stably or transiently transfected in HEK293 cells. This tag is highly detectable with an anti VSV-G antibody labeled with the CypHer 5 fluorochrome which is able to monitor the trafficking of the receptors from the cell surface into acidic endosomal pathways in live cells [28], [29]. This dye is pH-sensitive and fluorescent only in acidic environments (endosomes), but is non fluorescent at a neutral pH (cell surface). Compared to most common methods such as green fluorescent proteins tags [31], [32], this approach has the advantage of avoiding the fluorescent signal of membrane receptors. Given this particular property, the intracellular fluorescence corresponding to surface VSV-G tagged-receptor internalization could be assessed by real-time live cell imaging.

**Figure 3:**
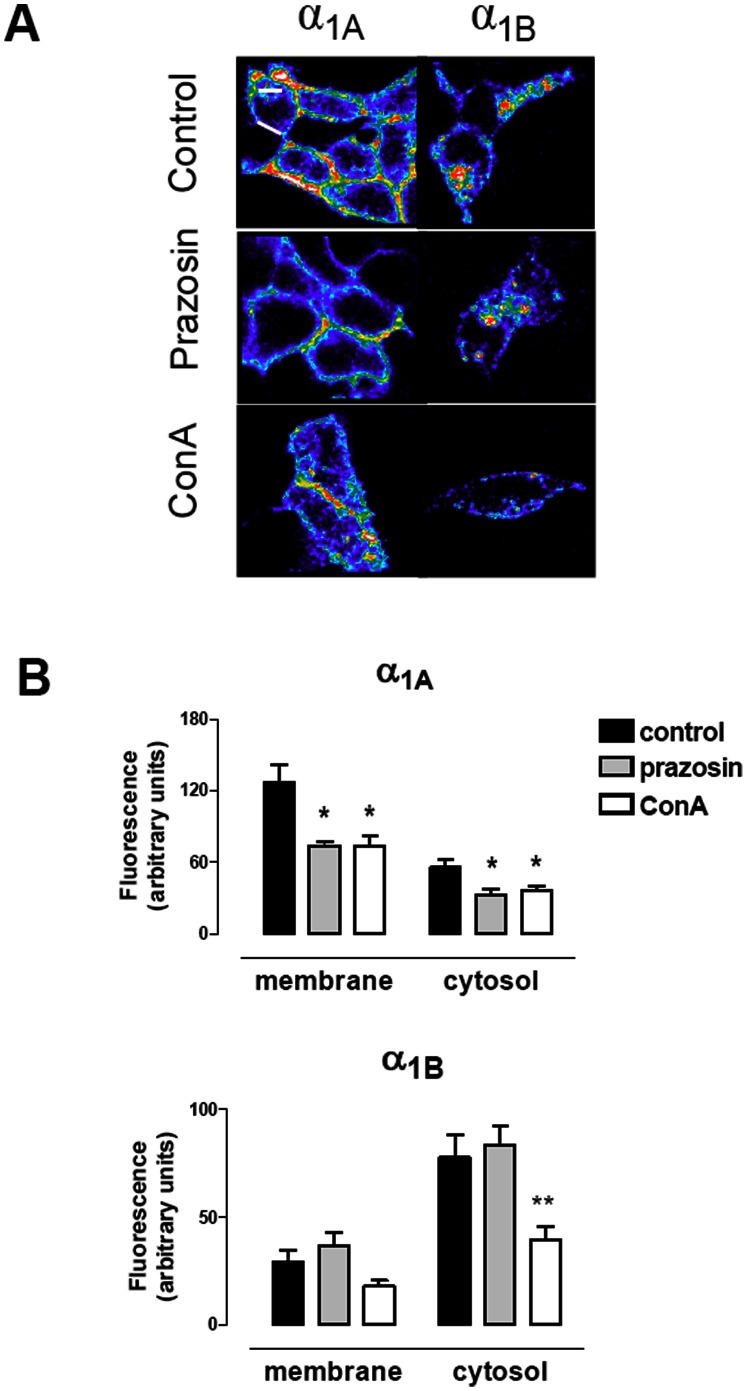
Quantitative analysis of the constitutive internalization of VSV-G α_1A_- and VSV-G α_1B_-ARs. Live HEK293 cells stably transfected with each subtype were incubated with CypHer5E Linked anti-VSV-G Antibody at a 5 µg/ml in KRH buffer at 4°C for 1h. After washing with cold KRH Buffer, coverslips were rapidly mounted into a chamber bath, placed on the confocal microscope stage in a 95% air and 5% CO_2_ atmosphere at 37°C. At this time, HEK293 were then exposed to prewarmed KRH buffer for 30 min at 37°C and the images were acquired. In some experiments 10 µM prazosin or 250 µg/ml concanavalin A (ConA) were added during the incubation time. (**A**) Confocal images representatives of the increase of intracellular fluorescence after 30 min of incubation at 37^a^C for both VSV-G-α_1A_- and VSV-G-α_1B_-ARs (**B**). Changes in intracellular fluorescence intensity were measured in the cells treated or not with prazosin or ConA. Intracellular fluorescence was quantified in the regions close to the cytoplasmic side of the plasma membrane, and in the cytosolic region away from nuclei (Figure S1). Data were expressed as arbitrary units of fluorescence and represent the mean ± S.E.M. of at least 4 independent experiments. Student’s *t* test: *p<0.05 **p<0.01 *vs* control.

Visualizing the Cypher 5 fluorescence in real-time demonstrates that the α_1A_ and the α_1B_ subtypes spontaneously internalize by endocytosis when expressed stably (Figure 3) or transiently (Figure S2) in HEK 293 cells. The experimental procedure needed to show this constitutive internalization is based on the incubation of live cells with CypHer 5 for 60 min at 4°C. At this temperature, the endocytic mechanism is blocked since the endocytosis pathway has been reported to be temperature-dependent [33], thus only the surface receptor will be labeled during this period.

After incubation with the CypHer5E linked Anti-VSV antibody at 4°C for 60 min to label the cell surface receptors, the fluorochrome was removed and live cells were rapidly mounted in a chamber bath linked to a confocal microscope. At this time the lack of a fluorescent signal (Figure S3) indicates that, at 4°C, endocytic mechanisms are blocked and, therefore, there is a lack of constitutive internalization [34] in stably transfected cells. Cells were then exposed to KRH buffer at 37°C for 30 min to allow equilibration. At this time point, the first image was acquired and the observed fluorescence (Figure 3) could be attributed to constitutive internalization of VSV-G-α_1A_-AR and VSV-G-α_1B_-AR. Fluorescence was not observed in non transfected cells (Figure S3). However the internalized receptor subtypes differed in their subcellular distribution as shown by the pattern of fluorescence. VSV-G-α_1A_-AR exhibited intracellular punctuate fluorescence distributed mainly in regions near the plasma membrane but with some of the receptors close to the cell nucleus (Figure 3A). By contrast VSV-G-α_1B_-AR was concentrated in clusters more homogeneously distributed throughout the cytosol. Quantification of the fluorescence confirmed the existence of two different regions (near to the plasma membrane and cytosolic) (Figure 3B) which could be interpreted as two differently located acidic endosomal pools. Similar results were found in transiently transfected cells (Figure S2)

Preincubation with the selective α_1_-AR antagonist/inverse agonist ligand prazosin (PZ) resulted in decreased localization of VSV-G-α_1A_-AR in both near-membrane and cytosolic regions, whereas it did not alter significantly the distribution pattern of the VSV-G-α_1B_-AR (Figure 3B). The clathrin-mediated endocytosis blocker concanavalin A (ConA) inhibited constitutive internalization of the VSV-G-α_1A_-AR to the near-membrane and cytosolic region, and also trafficking to the cytosolic region of the VSV-G-α_1B_-AR.

**Figure 4:**
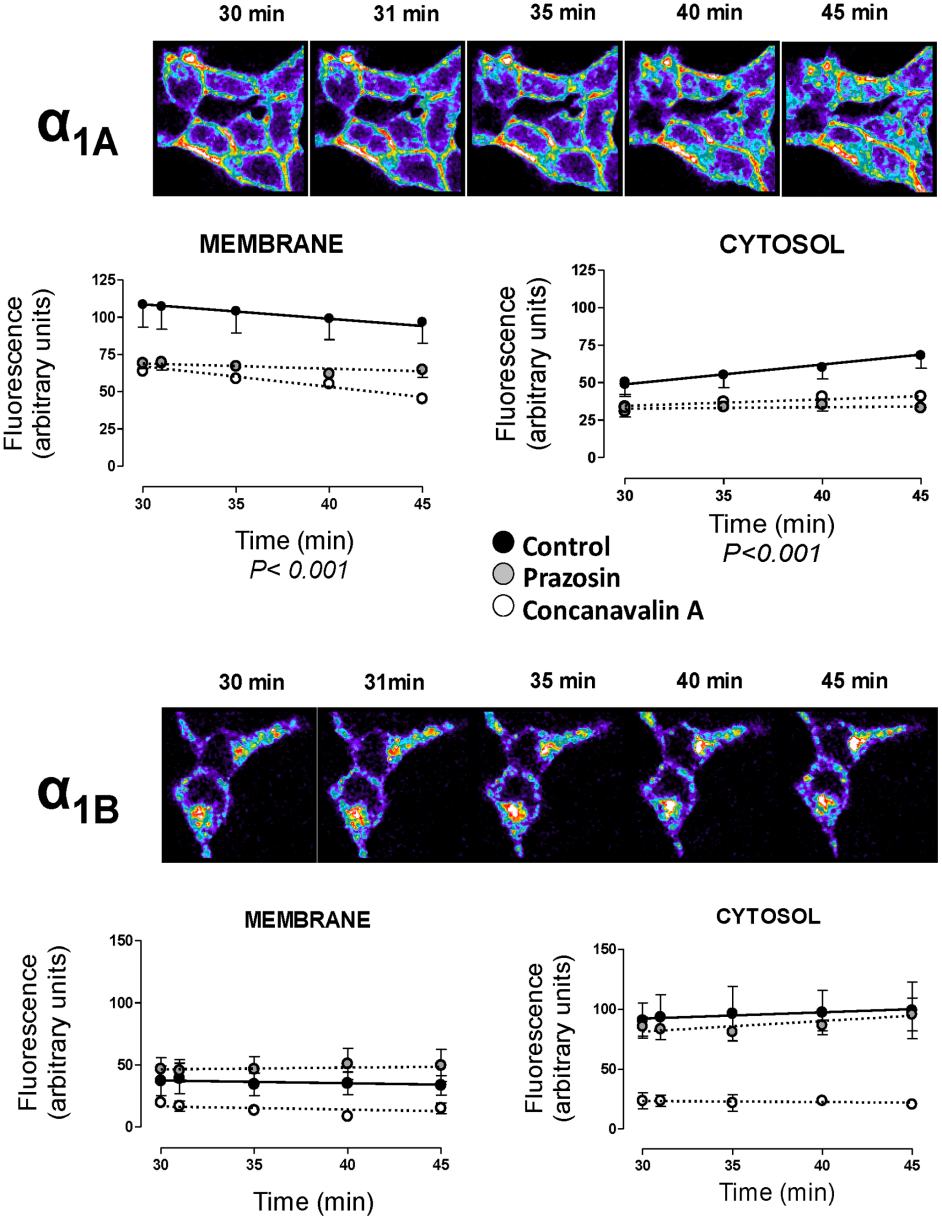
Spatio-temporal analysis of the constitutive internalization of VSV-G α_1A_- and VSV-G α_1B_-ARs. Live HEK293 cells stably transfected with each subtype were treated according to protocol detailed in Figure 3 and coverslips were rapidly mounted into a chamber bath, placed on the confocal microscope stage in a 95% air and 5% CO_2_ atmosphere at 37°C. At this time, HEK293 were then exposed to pre-warmed KRH buffer for 30 min. After 30 min of incubation, the images were acquired at zero time, 1 min and then 5 min intervals for 15 min. Confocal images are representatives of the increase of intracellular fluorescence for both VSV-G-α_1A_-AR and VSV-G-α_1B_-AR. Graphs represent the changes in intracellular fluorescence intensity over time in the regions close to the cytoplasmic side of the plasma membrane and in the cytosolic region away from nuclei in control experiments (black symbols), cells preincubated with 10 µM prazosin (grey symbols) or 250 µg/ml concanavalin A (white symbols). Data were expressed as arbitrary units of fluorescence and represent the mean ± S.E.M. of at least 4 independent experiments. Two-way ANOVA indicates a significant (p<0.001) time dependent change for α_1A_- membrane and cytosolic pools.

These spatial differences suggest specific patterns of constitutive endosomal trafficking for each subtype at this time point and were corroborated with the kinetic analysis of this constitutive internalization over time (Figure 4), by acquiring images in presence and absence of PZ or ConA at 35, 40 and 45 minutes after the equilibration period (30 min).

The constitutive internalization of the α_1A_ subtype continues until 45 min showing a significant decrease in the fluorescent intensity in the region near to the plasma membrane together with an increase in fluorescence in the cytosolic region over time (Figure 4). At the same time point, the VSV-G-α_1B_-AR did not exhibit an increased internalization suggesting that this process has reached its equilibrium during the initial time (30 min) needed to stabilize the system from 4° to 37°C. Incubation with prazosin or ConA maintains the differences observed at zero time without any significant change during the latter 15 min of incubation (from 30 to 45 min, Figure 4).

### Agonist-induced internalization to endosomes is subtype-specific and differently modulated by prazosin and ConA

**Figure 5:**
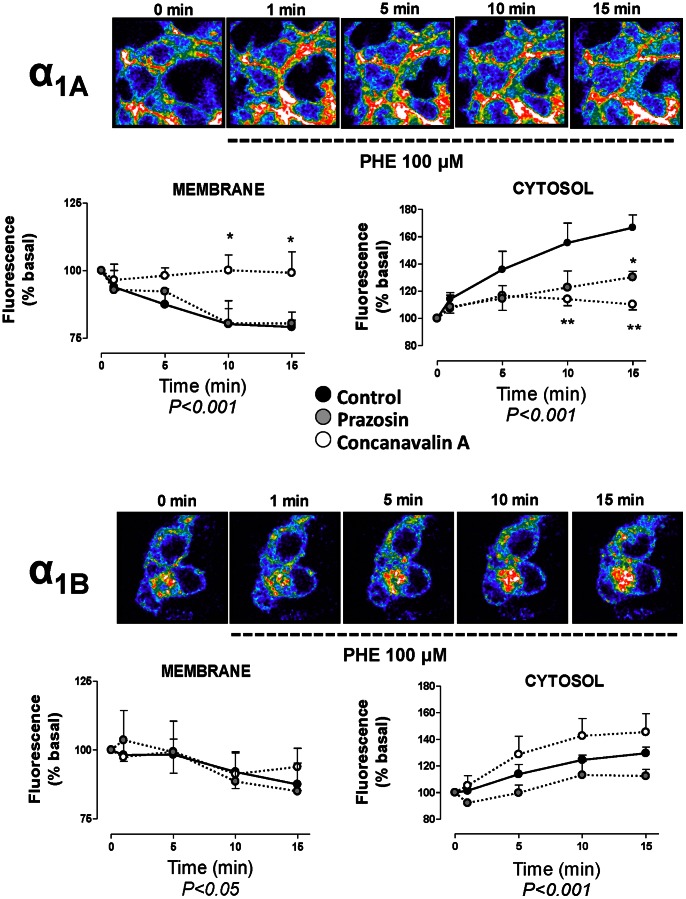
Spatio-temporal analysis of the agonist-induced internalization of VSV-G α_1A_- and VSV-G α_1B_-ARs. Live HEK293 cells stably transfected with each subtype were treated according to protocol detailed in Figure 3 and coverslips were rapidly mounted into a chamber bath, placed on the confocal microscope stage in a 95% air and 5% CO_2_ atmosphere at 37°C. After 30 min of incubation PHE 100 µM was added and the images were acquired immediately before PHE addition (zero time) and 1, 5, 10 and 15 min. Confocal images are representatives of the increase of intracellular fluorescence for both VSV-G-α_1B_-AR and VSV-G-α_1B_-AR. Graphs represent the significant changes in intracellular fluorescence intensity over time in the regions close to the cytoplasmic side of the plasma membrane and in the cytosolic region away from nuclei in control experiments (black symbols), cells preincubated with 10 µM prazosin (grey symbols) or 250 µg/ml concanavalin A (white symbols). Data were expressed as percentage of basal fluorescence determined before agonist addition (0 min) and represent the mean ± S.E.M. of at least 4 independent experiments. Two way ANOVA indicates a significant (p<0.001 or p<0.05) ) time dependent change for α_1A_ - membrane and cytosolic pools and for α_1B_-cytosolic pool. Student’s *t t*est was applied to determine significant differences at a given time vs control, *p<0.05 **p<0.01.

The addition of a α_1_-adrenoceptor agonist resulted in an increase in fluorescence over time compared to the untreated cells and induced different temporal patterns of endosomal internalization depending on the α_1_-AR subtype. As Figure 5 shows, incubation with PHE 100 µM intensifies the internalization process toward the intracellular regions close to the nuclei in both subtypes. When we quantified the cytosolic internalization corresponding to each VSV-G-α_1_-AR subtype stably transfected in the presence of PHE we observe a continuous increase in the fluorescence over the 15 min after PHE addition. Similar results were obtained in transiently transfected cells (Figure S4).

Agonist-mediated internalization was modulated by Prazosin and ConA. Prazosin 10 µM did not affect the PHE-induced internalization to the near-membrane or cytosolic endosomal pools of the VSV-G-α_1B_-ARs, nor the near-membrane internalization corresponding to the VSV-G-α_1A_-AR. However, the increase in the cytosolic fluorescence observed after PHE addition to the VSV-G-α_1A_-AR was significantly inhibited by Prazosin. Con A decreased the fluorescent signals corresponding to the membrane or cytosolic internalization of the VSV-G-α_1A_-ARs but did not inhibit agonist-mediated VSV-G-α_1B_ internalization to either of the endosomal regions (Figure 5).

### Temporal pattern of the cytosolic Ca^2+^ signal mediated by activation of α_1_-AR is subtype specific

**Figure 6:**
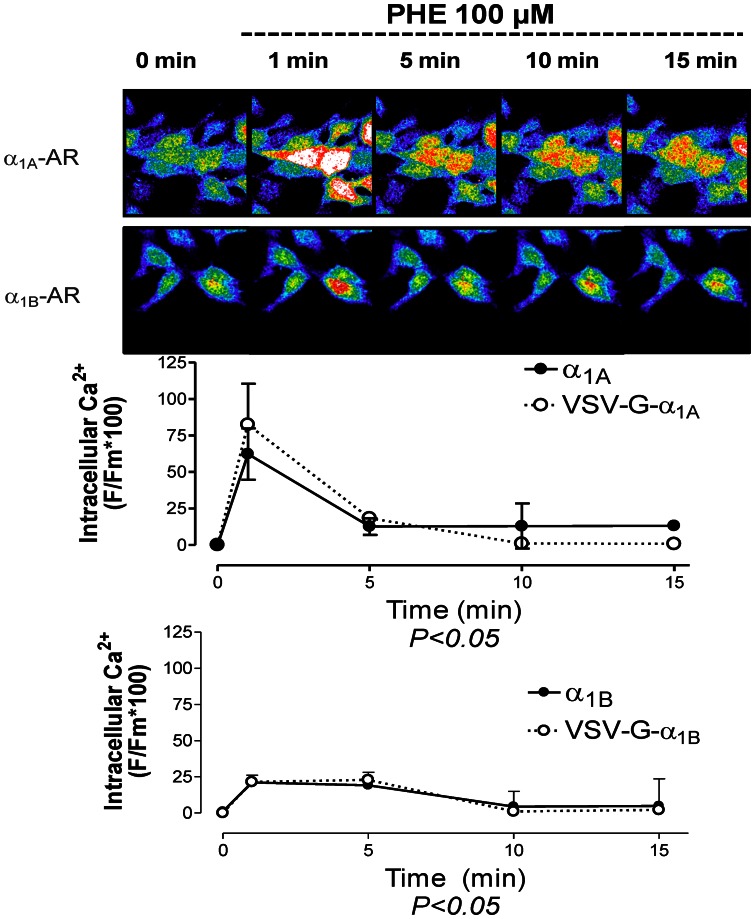
Activation of α_1_-ARs in cells stably transfected with the VSV-tagged or untagged α_1A_- and α_1B_-subtypes increases intracellular Ca^2+^. Live HEK293 cells stably transfected with each subtype were incubated for 2 h with the fluorescent Ca^2+^ quelant FLUO-3-AM and mounted into a chamber bath placed on the confocal microscopy stage in a 95% air and 5% CO_2_ atmosphere at 37°C. After 30 min of stabilization, images were collected before (zero time) and after (1, 5, 10 and 15 min) adding PHE 100 µM. Graphs represent the time-course of intracellular Ca^2+^ increase induced by α_1_-AR activation of VSV-tagged or untagged α_1A_- and α_1B_-ARs. The data were calculated as the increase in fluorescence observed after PHE addition over time, after substraction of the fluorescence observed in parallel experiments performed in the presence of PHE + prazosin (10 µM). Data represent the means ± S.E.M. of 5–8 independent experiments. Two way ANOVA indicates that activation of the VSV tagged and untagged α_1A_ and α_1B_-subtypes elicits a significant (p<0.05) time dependent change in the calcium signal.

Since α_1_-ARs couple to Gq proteins in the membrane to promote intracellular calcium elevation, cells transfected with the VSV-tagged or untagged α_1A_ and α_1B_ ARs were incubated with Fluo3-AM 5 µM to visualize alterations in the calcium-concentration. Representative confocal images are shown in Figure 6 where fluorescence intensities correspond with relative intracellular Ca^2+^ levels before (0 min) and after addition of PHE 100 µM (1, 5, 10 and 15 min) in HEK 293 cells stably transfected with the VSV-tagged α_1A_ and α_1B_ ARs.

PHE 100 µM evoked a rapid, time-dependent increase in the concentration of cytosolic-free calcium in HEK293 cells stably transfected with VSV-tagged or untagged α_1A_ and α_1B_ ARs which has a subtype specific temporal pattern (Figure 6). α_1A_-AR activation by PHE elicited a fast calcium signal which returned almost to basal levels after 5 min of PHE incubation. Similar results had been previously found in Rat-1 fibroblast stably expressing bovine α_1A_ adrenoceptors [35]. The α_1B_ transfected cells underwent an increase in intracellular calcium sustained until 10 min after PHE addition (Figure 6). PHE did not elicit any significant change in the fluorescence in presence of prazosin 10 µM (data not shown). Thus, the amino terminal VSV-G tag insertion in α_1_-ARs not modified the intracellular calcium signaling coupled to activation of each α_1_-AR subtype.

### α_1A_- and α_1B_-ARs signal through pERK 1/2 and p38-MAPK in a subtype specific manner

**Figure 7:**
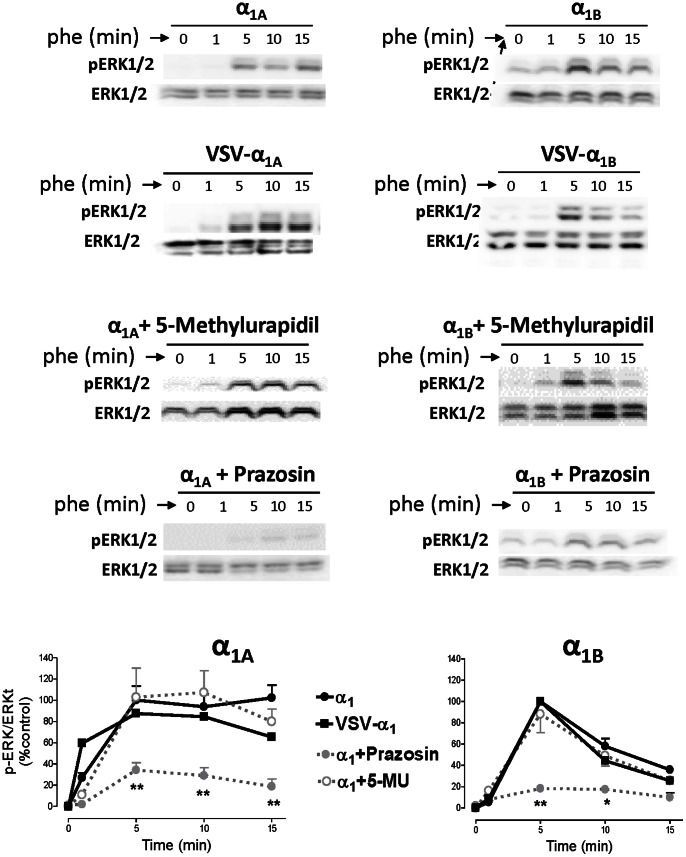
α_1A_- and α_1B_-AR stimulation shows a subtype-specific pattern of ERK1/2 phosphorylation. HEK293 cells stably transfected with VSV-tagged or untagged α_1A_- and α_1B_-subtypes were serum-starved for 4 hours and stimulated with PHE (100 µM) for a 15 minute time-course at 37°C. In some experiments, prazosin (10 µM) or 5-methylurapidil (10 µM) were added for 30 min. After stimulation, cellular extracts were prepared as described under the “Experimental procedures”. Equal amounts (15 µg) of each sample were used to visualize the p-ERK1/2 expression Western blots from representative experiments were shown. The lower panels show total ERK1/2 loaded on each sample. The graph quantifies the p-ERK1/2 signal at different times. Data represent means ± S.E.M. of 4-6 independent experiments. Student’s *t t*est was applied to determine significant differences at a given time vs control, *p<0.05 **p<0.01.

ERK1/2 activation in HEK 293 cells stably transfected with VSV-tagged or untagged α_1A_ and α_1B_ ARs was promoted by PHE 100 µM and completely blocked by prazosin 10 µM but not by 5-methylurapidil 10 µM (Figure 7). The ERK1/2 phosphorylation induced by each subtype followed a different temporal pattern. α_1A_-AR activation by PHE rapidly elicited a p-ERK1/2 signal (peak 1-5 min), which was well maintained for 15 min. The α_1B_ transfected cells underwent a similar initial increase in p-ERK1/2 but this was transient and the signal was rapidly reduced to basal level 10 min after PHE addition (Figure 7).

**Figure 8:**
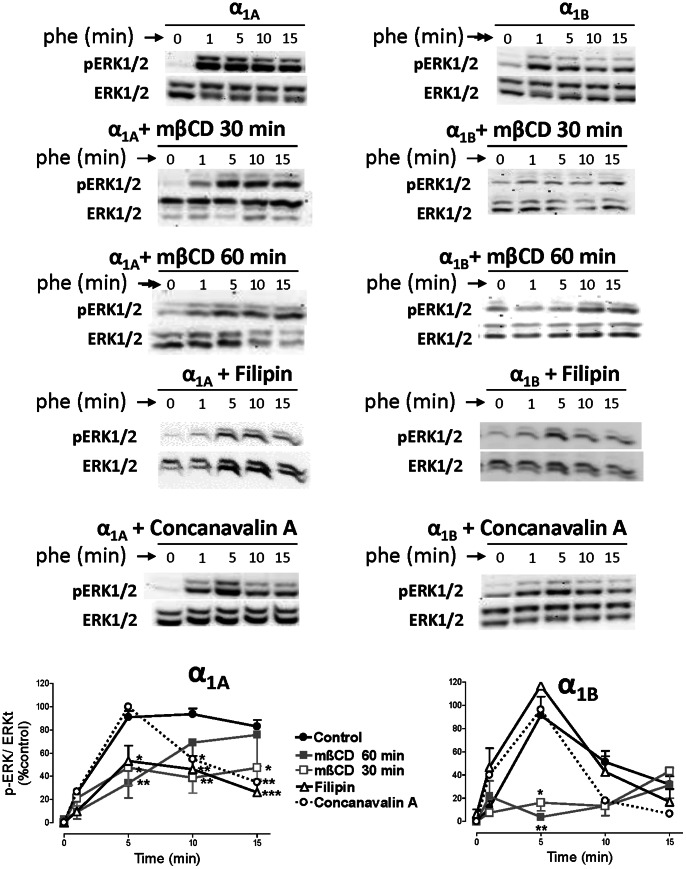
α_1A_- and α_1B_-AR mediated ERK1/2 activation is modulated by methyl-β-cyclodextrin, filipin and concanavalin A. HEK293 cells stably transfected with **α_1A_-** and α_1B_-AR subtypes were serum-starved for 4 hours and stimulated with PHE (100 µM) for a 15 minute time-course at 37°C. In some experiments, methyl-β-cyclodextrin 10 mM (mβCD) was added for 30 or 60 min, filipin 1 µg/ml and concanavalin A 250 µg/ml for 30 min. After stimulation, cellular extracts were prepared as described under the “Experimental procedures”. Equal amounts (15 µg) of each sample were used to visualize the p-ERK1/2 expression Western blots from representative experiments are shown. The lower panels show total ERK1/2 loaded on each sample. The graph quantifies the p-ERK1/2 signal at different times. Data represent means ± S.E.M. of 3-6 independent experiments. Student’s *t t*est was applied to determine significant differences at a given time vs control, *p<0.05 **p<0.01 ***p<0.001.

To assess the involvement of cholesterol-rich lipid rafts in regulation of α_1_-AR dependent ERK1/2 phosphorylation, cells stably transfected with α_1A_- or α_1B_- ARs were treated for 30 or 60 min (more or less energic conditions of membrane disruption) with mβCD 10 mM, or 30 min with filipin 1 µg/ml two reagents frequently utilized to disrupt lipid raft structure by depleting the cholesterol component. We also assayed the effect of Con A 250 µg/ml. As shown in Figure 8, the α_1A_-dependent p-ERK signal was partially inhibited by mβCD (30 and 60 min) and filipin. Con A did not affect the fast, but inhibited the slow and sustained ERK1/2 phosphorylation induced by the α_1A_-AR (Figure 8).

mβCD treatment for 30 or 60 min, eliminated the α_1B_-AR dependent ERK1/2 signal whereas Filipin and Con A treatment did not affect the ERK1/2 phosphorylation (Figure 8).

**Figure 9:**
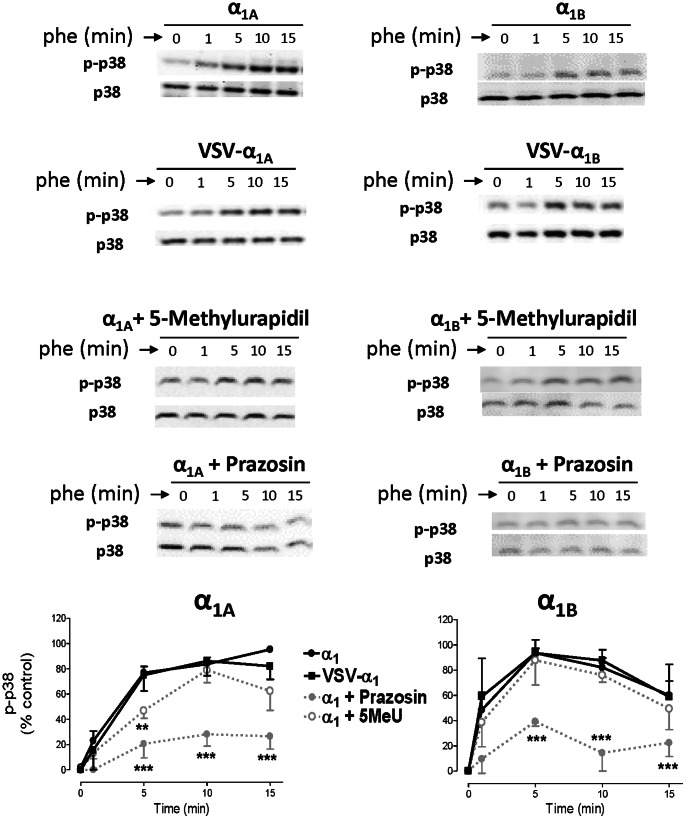
α_1A_- and α_1B_-AR stimulation shows a subtype-specific pattern of p38-MAPK phosphorylation. HEK293 cells stably transfected with VSV-tagged or untagged α_1A_- and α_1B_-subtypes were serum-starved for 4 hours and stimulated with PHE (100 µM) for a 15 minute time-course at 37°C. In some experiments, prazosin (10 µM) or 5-methylurapidil (10 µM) were added for 30 min. After stimulation, cellular extracts were prepared as described under the “Experimental procedures”. Equal amounts (15 µg) of each sample were used to visualize the phosphorylated p-38 MAPK expression Western blots from representative experiments are shown. The lower panels show total p38 loaded on each sample. The graph quantifies the p-p38 MAPK signal at different times. Data represent means ± S.E.M. of 3-6 independent experiments. Student’s *t t*est was applied to determine significant differences at a given time vs control, *p<0.05 **p<0.01 ***p<0.001.

PHE 100 µM promoted phosphorylation of the p38-MAPK in HEK 293 cells stably transfected with VSV-tagged or untagged α_1A_ and α_1B_ ARs. Figure 9 shows the p38-MAPK phosphorylation induced by each subtype which follows a different temporal pattern: the response appears and disappears more slowly when the α_1A_ subtype was activated. In both subtypes this response was abolished by prazosin 10 µM. The selective α_1A_- ligand 5-Methylurapidil only slowed the signal promoted by α_1A_-AR activation and did not affect the α_1B_-mediated p-p38 signal (Figure 9).

**Figure 10:**
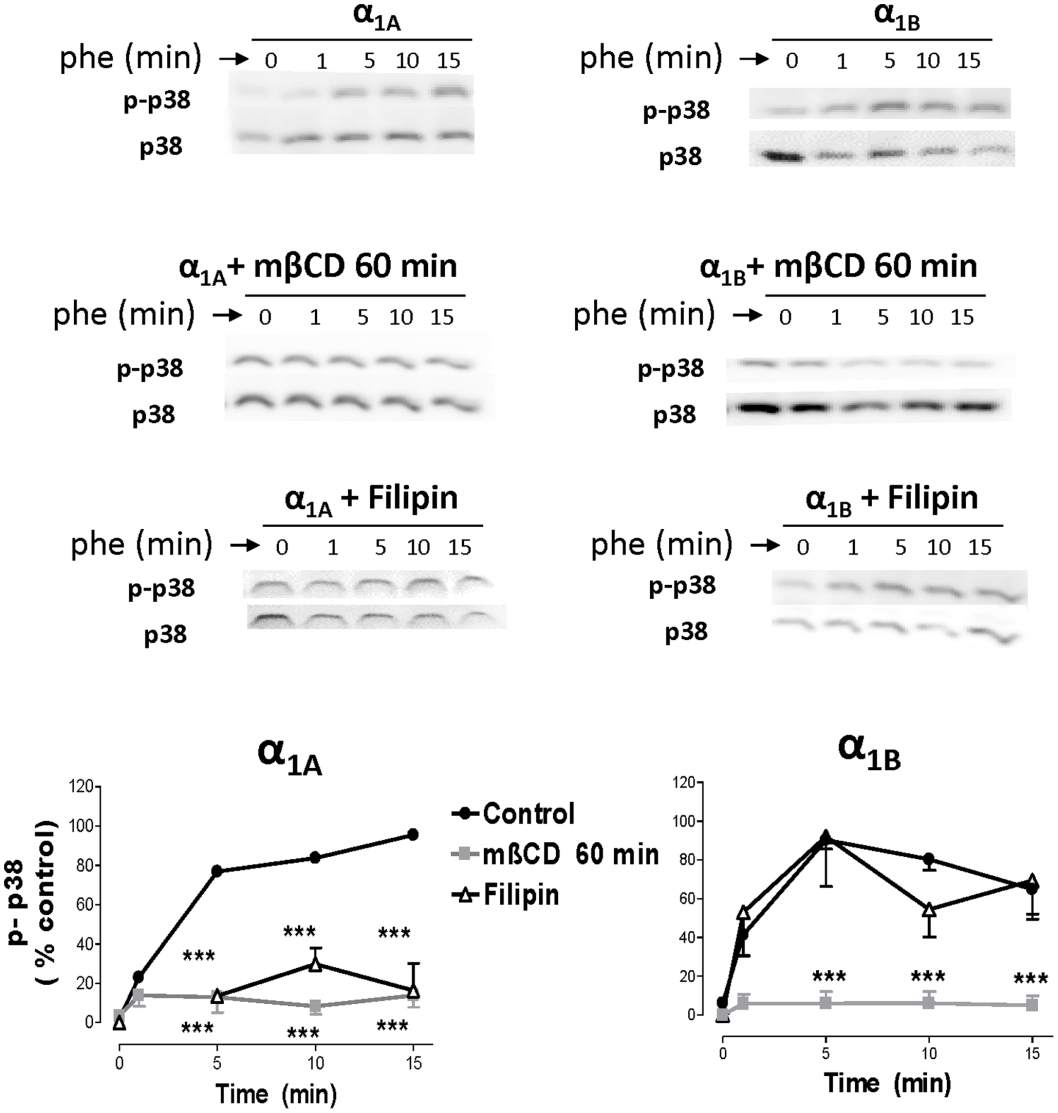
α_1A_- and α_1B_-AR mediated p-38 activation is modulated by methyl-β-cyclodextrin, filipin and concanavalin A. HEK293 cells stably transfected with each α_1_-AR subtype were serum-starved for 4 hours and stimulated with PHE (100 µM) for a 15 minute time-course at 37°C. In some experiments, methyl-β-cyclodextrin 10 mM (mβCD) was added for 30 or 60 min, filipin 1 µg/ml and concanavalin A 250 µg/ml for 30 min. After stimulation, cellular extracts were prepared as described under the “Experimental procedures”. Equal amounts (15 µg) of each sample were used to visualize the p-p38-MAPK expression Western blots from representative experiments are shown. The lower panels show amounts of p38-MAPK loaded on each sample. The graph quantifies the p-p38-MAPK signal at different times after agonist addition. Data represent means ± S.E.M. of 3–4 independent experiments. Student’s *t t*est was applied to determine significant differences at a given time vs control, ***p<0.001.

Pretreatment with mβCD 10 mM for 60 min abolished the p-p38 response induced by both subtypes. Filipin 1 µg/ml abolished α_1A_- but did not affect α_1B_-mediated p-p38 signal (Figure 10).

## Discussion

We present evidence for two major conclusions. The first is that, α_1A_- and α_1B_- ARs exhibit constitutive and agonist–induced endocytosis and are sorted to two different endosomal compartments, one of them located close to the inner face of the cell membrane whereas the other is deeper distributed in the cytosol. The other major finding is that each subtype was directed to these two endosomal pools with a different time-course pattern, and this peculiar distribution is closely related to the signalling pathway activated by each receptor, then to its functional response.

### Internalization patterns of α_1A_- and α_1B_-ARs

In absence of stimulus, VSV-tagged α_1_-ARs, previously located in the membrane, constitutively internalize into acidic endosomes located both, near the membrane or deeper in cytosol. This is confirmed by colocalisation with transferrin. After 30 min, the α_1A_ subtype was mainly distributed in endosomes located near the cytoplasmic side of the plasma membrane but this receptor pool decreases as time passes while, in contrast, the deeper cytosolic pool increases. This is confirmed by colocalisation with lysotracker. These observations made by new approaches agree with and extend previous findings about constitutive internalization of α_1A_-AR stably expressed in Rat-1 fibroblats [23], [27], but disagree with a previous study in which spontaneous internalization of the α_1A_ subtype transfected in HEK 293 cells has not been found [22]. In this case, differences could not be attributed to cell line or to the expression system since we found similar results in stable and transiently transfected cells, nor to the different receptor constructs, because they are different in all cases. The main reason to explain the discrepancies could be the method used to measure endocytosis: fluorescent ligands ([23], [27] and the present study) or biotinylation experiments [22]. Although receptor biotinylation is an accurate method to quantify receptors, it is possible that it does not detect small receptor movements such as α_1A_ ARs moving faster from the membrane to the endosomes located near the membrane.

The α_1B_ subtype also undergoes constitutive internalization, but with a different subcellular distribution. The fluorescent signal was concentrated as clusters homogeneously distributed within the cell, confirming previous works [22], but differing from Morris et al., [23] who did not find constitutive internalization in a preliminary experiment with HA-tagged α_1B_ EGFP ARs. When we quantified the intensity of the real-time fluorescence for 15 min more, we saw no changes in fluorescence in either of the regions measured (close to the cytoplasmic side of the plasma membrane or in the deeper cytosol).

Agonist addition intensified the internalization process especially for the α_1A_ subtype, which rapidly moves from the endosomes located close to the inner face of the membrane to the deeper endosomes.

Clathrin-mediated internalization could be involved in agonist-induced [18] as well as constitutive endocytosis of GPCRs [34], and constitutive internalization of α_1A_ has been described as a mechanism which depends on β-arrestin and clathrin [23], [27]. Our results support these findings as the spontaneous traffic of α_1A_- and α_1B_-ARs was partially inhibited by concanavalin A, a validated blocker of endocytosis mediated by clathrin-coated pit/vesicle formation [27]. Concanavalin A also inhibits agonist-mediated α_1A_-AR internalization as previously described [23], [27], although it did not affect α_1B_-AR agonist-mediated internalization. This observation contrasts with previous results which suggested that agonist stimulation of α_1B_-AR endocytosis is dependent on β-arrestin [22]. However, as we have already stated, the different methodological approaches could be the reason for such discrepancies. Prazosin modified constitutive (both near membrane and deeper pools), and agonist induced endocytosis (deeper pool) of the α_1A_ subtype but did not alter the spontaneous nor the agonist induced internalization of the α_1B_ subtype. Similar results have been reported for the α_1A_- subtype, using the HA-tagged α_1A_-AR [23], [25] or the fluorescent prazosin-derivative QAPB [27]. The surprising lack of a significant effect of prazosin on the internalization of the α_1B_-subtype has not been previously described.

### Comparison between Ca^2+^-signal, activation of ERK1/2 or p38-MAPK and the endocytic pathways of the α_1_-AR subtypes

**Figure 11:**
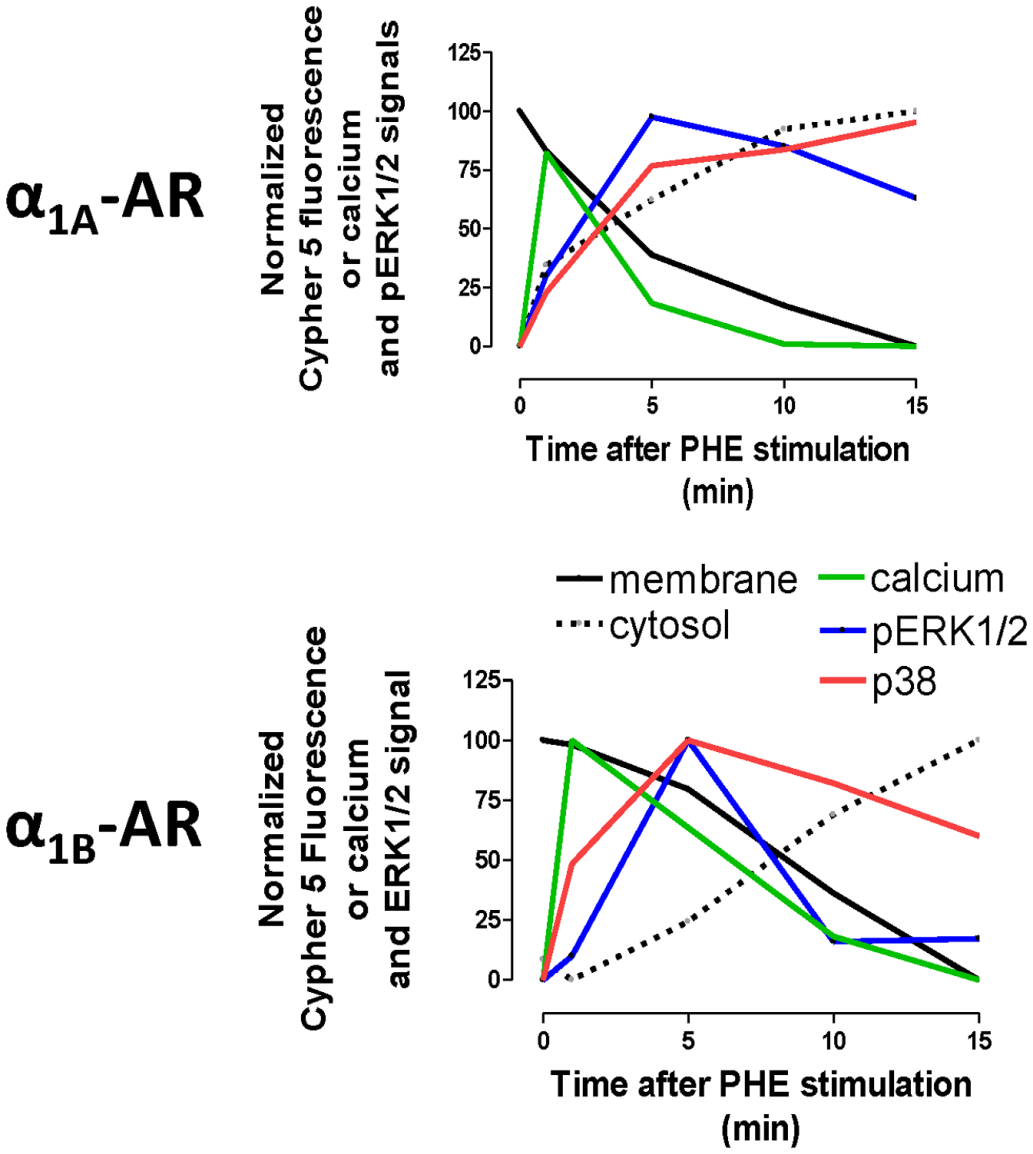
Combined analysis of the temporal patterns of calcium signal, pERK1/2 signal and internalization of VSV-α_1_-ARs following stimulation. Schematic diagram representing the kinetic of the internalization patterns of VSVG-α_1A_ and VSVG-α_1B_ ARs in endosomes located near the plasma membrane (continuous line) or in the cytosol (discontinuous line), and the kinetic of the intracellular calcium signal (green), or the pERK1/2 signal (blue) elicited by activation of these receptors or by activation of the receptors after disruption of lipid rafts with mβCD (red). In order to facilitate the comparison, the data were calculated as a percentage of the maximal response in each case, and represent the mean of the experiments previously described

After PHE stimulation of VSV-tagged or untagged α_1A_- and α_1B_-ARs, a strong increase in the calcium levels was observed. It is well-known that receptor endocytosis can attenuate or terminate membrane-located cell signaling. In our case, the strong increase in the calcium signal starts to decline together with a decrease in receptor localization in the near-membrane pool of endosomes. A close parallel was observed between the passage to the deeper endosomes and disappearance of the calcium signal and finally, when α_1_-AR localization in the deeper endosomes is achieved at this maximal level, the localization in the near-membrane pool is minimal and the calcium concentration is close to the basal levels even in the continued presence of PHE (Figure 11). In this case, it seems that receptor endocytosis from membrane to deeper endosomes terminates cell calcium-signaling. This observation agrees with the well-known α_1_-AR signaling pathway which involves membrane located protein Gq and inositol phosphates. As well as α_1A_- or α_1B_- AR moving from the membrane to deeper endosomes, the coupling with the Gq protein is interrupted and calcium signal declines.

pERK1/2 kinetics elicited by PHE-activation of α_1A_ and α_1B_-ARs, gave a different pattern for each subtype. α_1B_-induced ERK1/2 phosphorylation, which reaches its maximum 5 min after PHE stimulation and decreases at the same time as α_1B_-ARs located near the membrane disappear (Figure 11). As expected, this signal was completely inhibited by prazosin but not by the selective α_1A_ ligand 5-methylurapidil. To investigate in detail the importance of the membrane integrity for receptor induced signaling we use two agents frequently utilized to disrupt lipid raft structure by depleting the cholesterol component: mβCD and filipin. However, the effect of these agents on the membrane integrity is not identical. In fact, filipin treatment completely changes the cellular distribution of caveolin-1 leading to a collapse of the caveolae, whereas mβCD sequestrates cholesterol without any significant effect on caveolin-1 distribution [36]. As α_1B_ mediated ERK1/2 phosphorylation was not observed after membrane disruption by mβCD treatment (30 or 60 min) but remained unaltered after filipin treatment, we can assume that caveolae, important for internalisation of GPCR, are not essential components of α_1B_-AR signaling pathway, which depends on membrane integrity. This result agrees with the observation that the clathrin-mediated endocytosis blocker concanavalin A [27] does not modified the ERK1/2 signal activated by the α_1B_ -subtype.

A different ERK1/2 response was observed with the α_1A_-AR. This subtype elicited a rapid ERK1/2 phosphorylation inhibited by membrane and caveolae disruption but insensitive to Concanavalin A treatment, indicating its dependence of membrane integrity but not to clatrhin-mediated internalizacion, which is in agreement with previous reports showing that agonist elicited α_1A_-dependent pERK1/2 signal depends on caveolae integrity [25], [37] In addition, concomitant with phenylephrine induced receptor internalization, the α_1A_-subtype produced a maintained ERK phosphorylation, which was inhibited by concanavalin A. This later signal did not disappear over 15 min and we found high levels of pERK1/2 even when the α_1A_ localization in the deeper endosomes has reached its maximum (Figure 11). The existence of a remaining pERK1/2 signal which depends on endocytosis has previously been proposed for α_1A_-ARs and other GPCRs [38], [39], [40], [41]. This late α_1A_-mediated pERK1/2, observed after lipid raft disruption by mβCD or caveolae disorganization by filipin, but inhibited by Concanavalin A, corroborates the existence of an ERK1/2 signal independent of the membrane or caveolae integrity but dependent on receptor internalization.

Our results also show that prazosin, an antagonist which freely crosses the plasma membrane [42] and internalizes with receptors [27], blocks the calcium signal as well as ERK1/2 and p38 phosphorylation elicited by PHE whereas 5-methylurapidil does not. Similar results were previously described in cardiomyocites where PHE-induced activation of ERK1/2 was blocked by prazosin but nor by the membrane impermeable α1 antagonist as CGP-12177A [42]

Previous evidence indicates that α_1A_-ARs, via ERK activation, exert an anti-apoptotic activity with a cardioprotective role in cardiomyocytes that was not evident for the other α_1_ subtypes [43]. The fact that the pool of receptors responsible for this anti-apoptotic activity was perinuclearly localized, strongly suggests that it is dependent on the receptor-internalization. Our present results suggest that the slow and sustained p-ERK activation induced by α_1A_-AR located in endosomes could be the prosurvival pathway relevant for cardioprotection, and could explain the clinical evidence that chronic use of a α_1_ antagonist as doxazosin was associated with higher risk factor for coronary heart disease [44]. The fact that ERK1/2 phosphorylation mediated by the α_1A_ subtype was completely inhibited by prazosin but not by the selective α_1A_ ligand 5-methylurapidil was an unexpected result that opens new perspectives to investigate the inhibitory profile of the α_1_-selective ligands on MAPKs signals to improve its therapeutic usefulness.

Finally, both α_1A_ and α_1B_-ARs phosphorylate p38 when activated by PHE and this phosphorylation was inhibited by prazosin. 5-methylurapidil only retards the p38 signal promoted by the α_1A_ subtype. The temporal pattern of p38 phosphorylation did not correlate to the other pathways analyzed. Activation of the α_1A_-AR elicits a slow phosphorylation of p38 entirely dependent on lipid rafts and caveolae integrity, suggesting that the signal is not related to the endocytic process, or if it is, only receptors internalized in intact lipid rafts can signal through this p38-MAPK pathway raft-dependent endocytosis [45]. Activation of the α_1B_-AR induces a faster p38-MAPK signal, which also depends on the lipid rafts integrity, since it was not observed after mβCD treatment, but was unaffected by filipin, confirming that maintenance of caveolae structure is not essential for α_1B_-signalling. In this case we cannot conclude if the activation of the p38-MAPK pathway by the α_1B_-AR depends or not, at least partially, on the receptor internalization process, but precedes it (Figure 11).

Controversial results provide evidence that activation of p38 can be beneficial or detrimental in a cell [46], but recent data showed that activation of α_1_-AR in cardiomyocytes induces p38 activation and has a protective role to antagonize noradrenaline-induced cell death [47]. Future studies about the functional consequences of the p38 activation by each α_1_-AR subtype could add more information about the cardioprotective role of α_1A_-ARs and the relationship between cardioprotection and the property of continuing signaling once the receptor has been internalized into the deeper cytosolic endosomes.

In short, constitutive as well as agonist-induced trafficking of α_1A_ and α_1B_ ARs maintain two different endosomal pools of receptors with different subcellular distribution: one located close to the plasma membrane and the other deeper in the cytosol. Each subtype exhibits peculiar characteristics of internalization and distribution between both pools that condition their signaling pathways: α_1A_-AR, when located in the plasma membrane, signal through calcium and ERK1/2 pathways but, when it translocates to deeper endosomes, continues signaling through ERK1/2 and could also activate the p38 pathway. α_1B_-AR signals through calcium, ERK1/2 and p38 only when located in the membrane and the signals disappear by membrane disruption. The functional consequences of the intracellular signaling could be related to the cardioprotective activity shown by the α_1A_ subtype.

## Supporting Information

Figure S1 **Internalization kinetic was quantified for each cell at two different cellular regions (cytosol and near-membrane) by measuring the mean intensity of the fluorescence of two linear segments of 5** µ**m of length located in the cytosol, close to the nucleus, and two linear segments of 5** µ**m of length located in regions near to the plasma membrane.** Data were the mean of the measures obtained from 8–10 different cells for each experiment.To better identify membrane and cytosolic regions, light microscopic images were overlapped with fluorescence images.
(TIFF)

Figure S2 **Live HEK293 cells transiently transfected with VSV-G-α_1A_- or VSV-G-α_1B_-AR subtypes were incubated with CypHer5E Linked anti-VSV-G Antibody at a 5** µ**g/ml in KRH buffer at 4°C for 1h.** After washing with cold KRH Buffer, coverslips were rapidly mounted into a chamber bath, placed on the confocal microscope stage in a 95% air and 5% CO_2_ atmosphere at 37°C. At this time, HEK293 were then exposed to prewarmed KRH buffer at 37°C. After 30 min of incubation the images were acquired at zero time (30 min), 31 min and then 5 min intervals for 15 min (45 min). Confocal images are representatives of the increase of intracellular fluorescence for both VSV-G-α_1A_-AR and VSV-G-α_1B_-AR.
(TIFF)

Figure S3 A) Live non transfected HEK293 cells were incubated with CypHer5E Linked anti-VSV-G Antibody at a 5 µg/ml in KRH buffer at 4°C for 60 min. After washing with cold KRH Buffer, coverslips were rapidly mounted into a chamber bath, placed on the confocal microscope stage in a 95% air and 5% CO_2_ atmosphere at 37°C. At this time, HEK293 were then exposed to prewarmed KRH buffer for 30 min at 37°C and the images were acquired (left: fluorescence; right: transmission) B) HEK293 cells stably expressing VSV-G-α_1A_ and VSV-G-α_1B_-AR subtypes after incubation with the CypHer5E linked Anti-VSV antibody at a 5 µg/ml in KRH buffer at 4°C for 60 min. Cells were washed with cold Krebs Ringer Buffer three times at 4°C, immediately, cells were fixed with 3.7%paraformaldehyde in PBS/4% sucrose for 10 min. Coverslips were mounted onto glass slides with Daco mounting medium and stored at 4°C in the dark until viewing in the confocal microscope. No significant fluorescence was observed under these conditions
(TIFF)

Figure S4 **Live HEK293 cells transiently transfected with VSV-G-α_1A_- or VSV-G-α_1B_-AR subtypes were treated according to protocol detailed in Figure b and coverslips were rapidly mounted into a chamber bath, placed on the confocal microscope stage in a 95% air and 5% CO_2_ atmosphere at 37°C.** After 30 min of incubation PHE 100 µM was added and the images were acquired immediately before PHE addition (zero time) and 1, 5, 10 and 15 min. Confocal images are representatives of the increase of intracellular fluorescence for both VSV-G-α_1A_-AR and VSV-G-α_1B_-AR.
(TIFF)
