# Supplementary figures and images for: Differences in the Signaling Pathways of α1A- and α1B-Adrenoceptors Are Related to Different Endosomal Targeting
Source: PLoS One. 2013 May 24;8(5):e64996. doi: 10.1371/journal.pone.0064996 (PMC3663791; doi:10.1371/journal.pone.0064996)

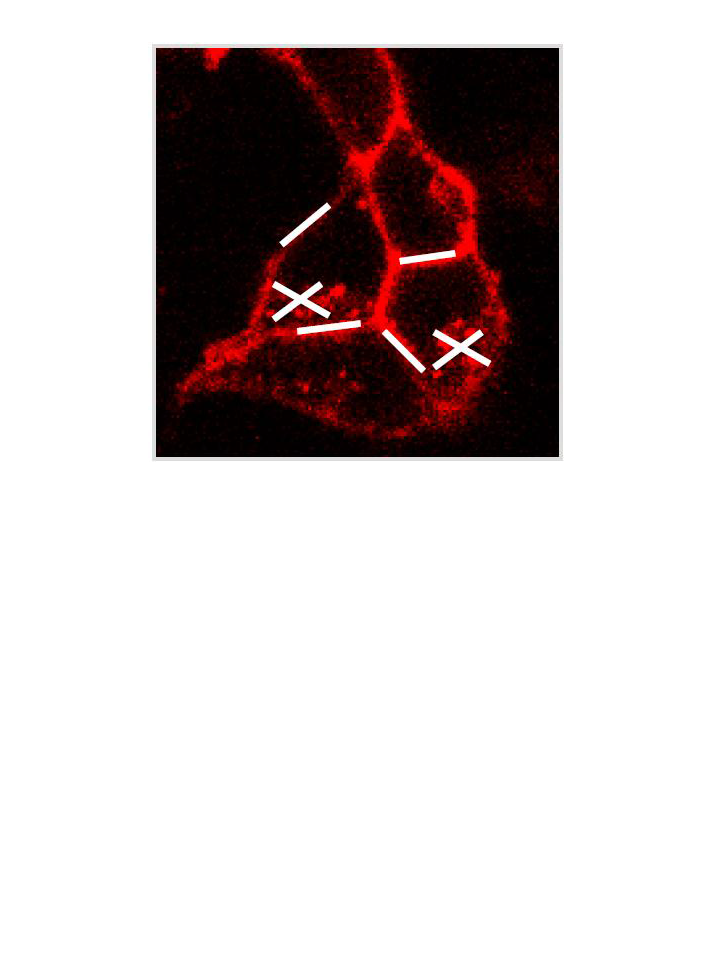

Supplement: Figure S1 — Internalization kinetic was quantified for each cell at two different cellular regions (cytosol and near-membrane) by measuring the mean intensity of the fluorescence of two linear segments of 5 µm of length located in the cytosol, close to the nucleus, and two linear segments of 5 µm of length located in regions near to the plasma membrane. Data were the mean of the measures obtained from 8–10 different cells for each experiment.To better identify membrane and cytosolic regions, light microscopic images were overlapped with fluorescence images. (TIFF) [file pone.0064996.s001.tif]

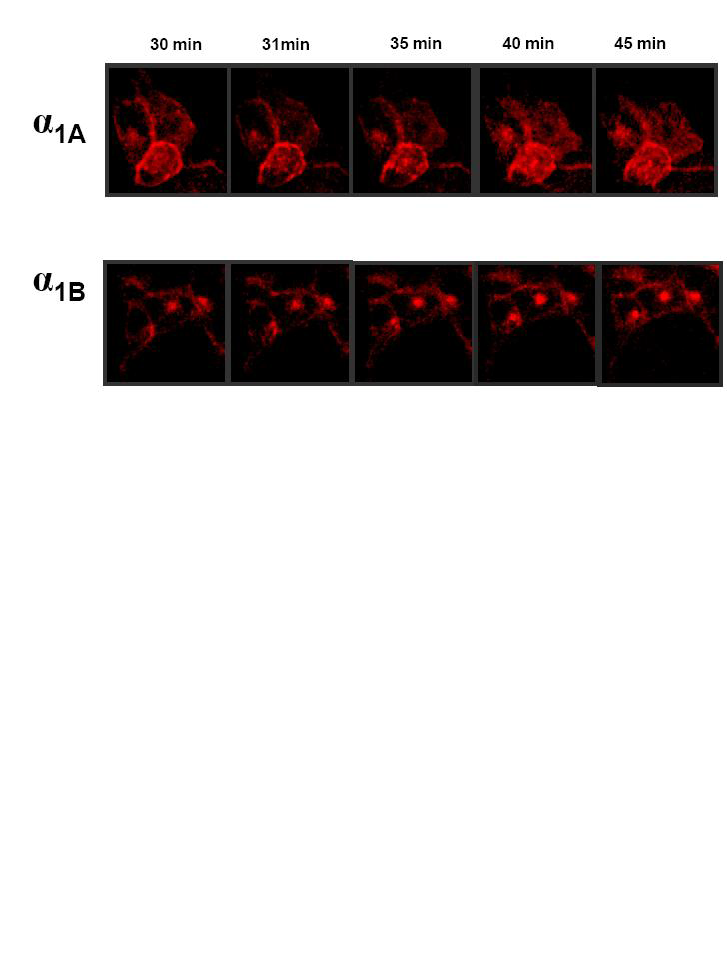

Supplement: Figure S2 — Live HEK293 cells transiently transfected with VSV-G-α1A- or VSV-G-α1B-AR subtypes were incubated with CypHer5E Linked anti-VSV-G Antibody at a 5 µg/ml in KRH buffer at 4°C for 1h. After washing with cold KRH Buffer, coverslips were rapidly mounted into a chamber bath, placed on the confocal microscope stage in a 95% air and 5% CO2 atmosphere at 37°C. At this time, HEK293 were then exposed to prewarmed KRH buffer at 37°C. After 30 min of incubation the images were acquired at zero time (30 min), 31 min and then 5 min intervals for 15 min (45 min). Confocal images are representatives of the increase of intracellular fluorescence for both VSV-G-α1A-AR and VSV-G-α1B-AR. (TIFF) [file pone.0064996.s002.tif]

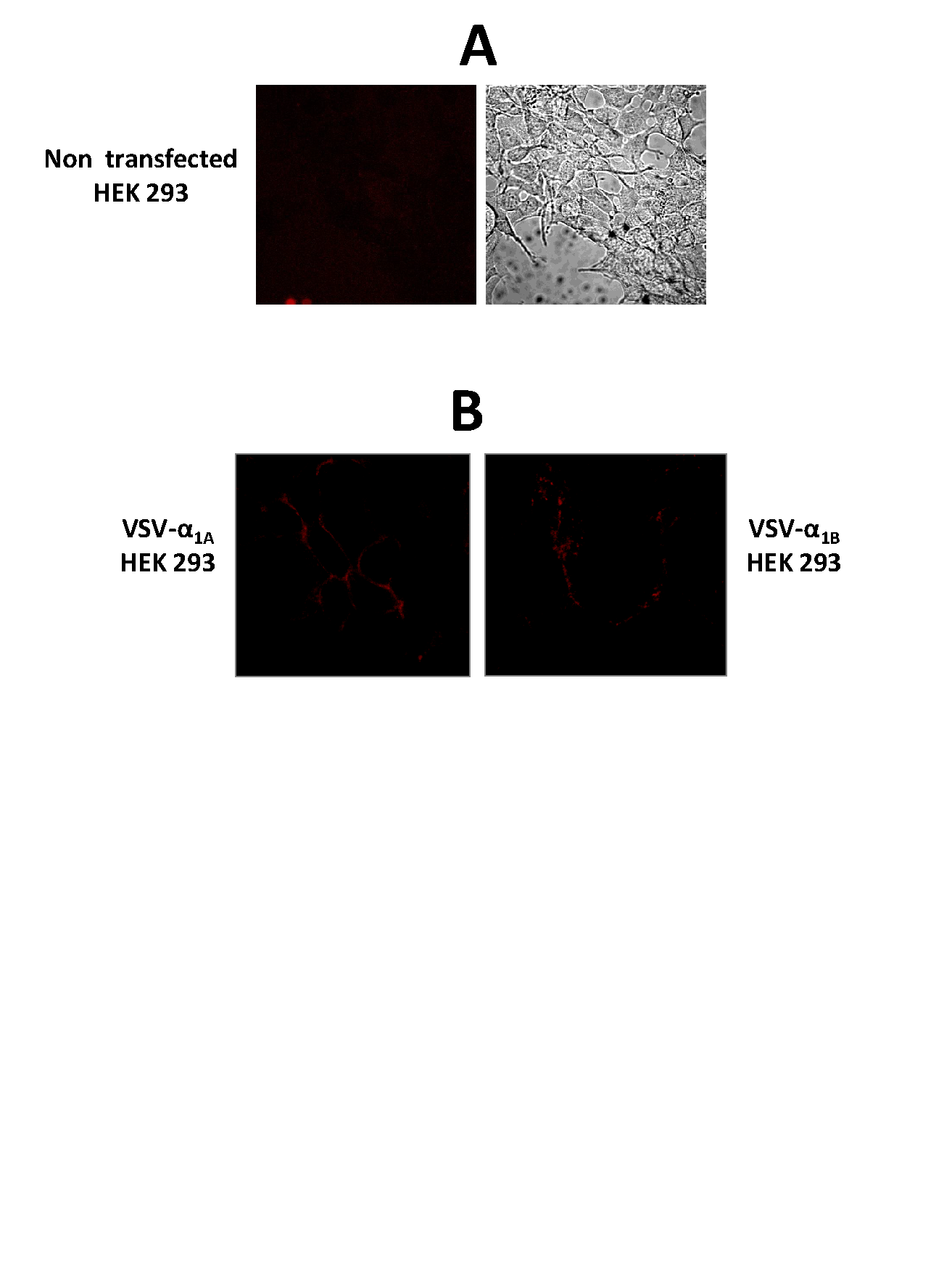

Supplement: Figure S3 — A) Live non transfected HEK293 cells were incubated with CypHer5E Linked anti-VSV-G Antibody at a 5 µg/ml in KRH buffer at 4°C for 60 min. After washing with cold KRH Buffer, coverslips were rapidly mounted into a chamber bath, placed on the confocal microscope stage in a 95% air and 5% CO2 atmosphere at 37°C. At this time, HEK293 were then exposed to prewarmed KRH buffer for 30 min at 37°C and the images were acquired (left: fluorescence; right: transmission) B) HEK293 cells stably expressing VSV-G-α1A and VSV-G-α1B-AR subtypes after incubation with the CypHer5E linked Anti-VSV antibody at a 5 µg/ml in KRH buffer at 4°C for 60 min. Cells were washed with cold Krebs Ringer Buffer three times at 4°C, immediately, cells were fixed with 3.7%paraformaldehyde in PBS/4% sucrose for 10 min. Coverslips were mounted onto glass slides with Daco mounting medium and stored at 4°C in the dark until viewing in the confocal microscope. No significant fluorescence was observed under these conditions (TIFF) [file pone.0064996.s003.tif]

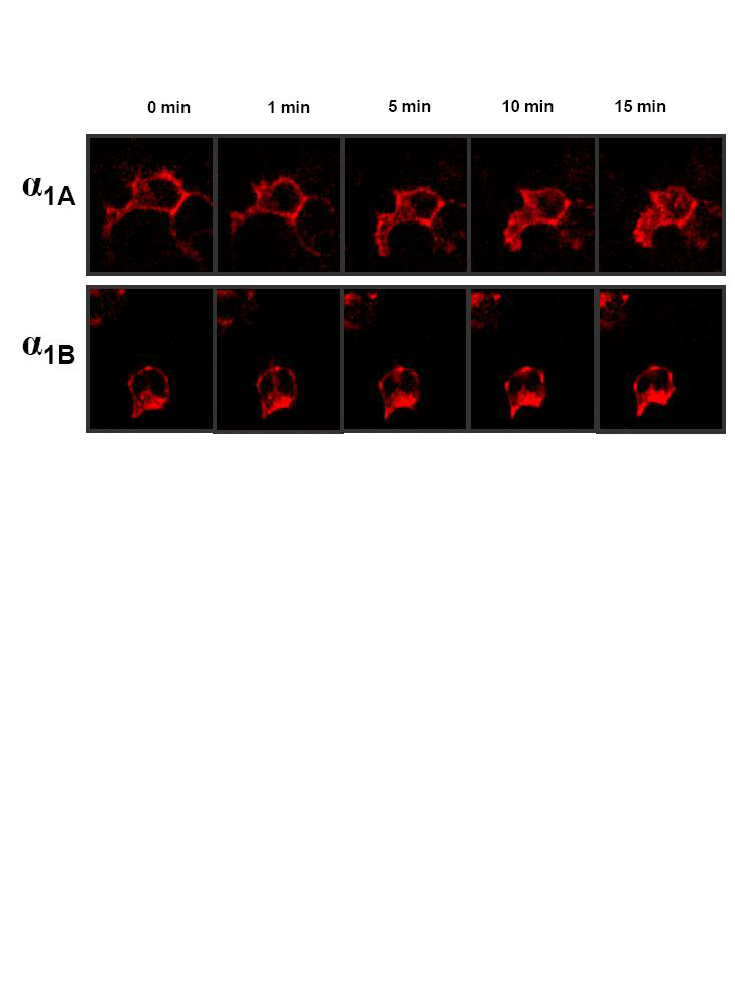

Supplement: Figure S4 — Live HEK293 cells transiently transfected with VSV-G-α1A- or VSV-G-α1B-AR subtypes were treated according to protocol detailed in Figure b and coverslips were rapidly mounted into a chamber bath, placed on the confocal microscope stage in a 95% air and 5% CO2 atmosphere at 37°C. After 30 min of incubation PHE 100 µM was added and the images were acquired immediately before PHE addition (zero time) and 1, 5, 10 and 15 min. Confocal images are representatives of the increase of intracellular fluorescence for both VSV-G-α1A-AR and VSV-G-α1B-AR. (TIFF) [file pone.0064996.s004.tif]
